# Genetic diversity of *Plasmodium falciparum* isolates based on MSP-1 and MSP-2 genes from Kolla-Shele area, Arbaminch Zuria District, southwest Ethiopia

**DOI:** 10.1186/s12936-015-0604-8

**Published:** 2015-02-14

**Authors:** Hussein Mohammed, Tedla Mindaye, Meseret Belayneh, Moges Kassa, Ashenafi Assefa, Mekonnen Tadesse, Adugna Woyessa, Tesfaye Mengesha, Amha Kebede

**Affiliations:** Ethiopian Public Health Institute, Addis Ababa, Ethiopia; Department of Laboratory Sciences, Addis Ababa University, Addis Ababa, Ethiopia

## Abstract

**Background:**

The genetic diversity of *Plasmodium falciparum* has been extensively studied in various countries. However, limited data are available from Ethiopia. This study was conducted to evaluate the extent of genetic diversity of *P. falciparum* in Kolla-Shele, in the southwest of Ethiopia.

**Methods:**

A total of 88 isolates from patients with uncomplicated *P. falciparum* attending Kolla-Shele Health Centre was collected from September to December, 2008. After extraction of DNA by Chelex® method, the samples were genotyped by using nested-PCR of *msp1* (block 2) and *msp2* (block 3) including their allelic families: K1, MAD20, RO33 and FC27, 3D7/IC1, respectively.

**Results:**

Allelic variation in both *msp1* and *msp2* were identified in the 88 blood samples. For *msp1* 67% (59/88) and *msp2* 44% (39/88) were observed. K1 was the predominant *msp1* allelic family observed in 33.9% (20/59) of the samples followed by RO33 and MAD20. Of the *msp2* allelic family 3D7/IC1 showed higher frequency (21.5%) compared to FC27 (10.3%). A total of twenty-three alleles were detected; of which, eleven were from *msp2* and twelve from *msp2* genes. Fifty-nine percent of isolates had multiple genotypes and the overall mean multiplicity of infection was 1.8 (95% CI: 1.48-2.04). The heterozygosity index was 0.79 and 0.54for *msp1* and *msp2*, respectively. There was no statically significant difference in the multiplicity of infection by either age or parasite density (P > 0.05).

**Conclusion:**

This genetic diversity study showed the presence of five allelic types in the study area, with dominance K1 in the *msp1* family and 3D7/IC1 in the *msp2* family. Multiple infections were observed in nearly 60% of the samples.

**Electronic supplementary material:**

The online version of this article (doi:10.1186/s12936-015-0604-8) contains supplementary material, which is available to authorized users.

## Background

Malaria, a disease mostly caused by *Plasmodium falciparum*, is a major public health problem. The global burden is 207 million malaria cases every year resulting into 627,000 deaths [1], sub-Saharan Africa being the most affected region. In Ethiopia, malaria still remains a major health problem with about two-thirds of the population living in malarious areas [2]. Among the malaria parasites, *P. falciparum* is the most fatal species, and exhibits complex genetic polymorphism which may explain its ability to develop multiple drug resistance and circumvent vaccines [3].

Recent, studies of anti-malarial drug resistance in Ethiopia have confirmed high level of resistance to sulphadoxine-pyrimethamine. Consequently, in 2004, Ethiopia changed its anti-malarial drug policy to artemisinin-based combination therapy (ACT) Coartem® as first-line drug for the treatment of uncomplicated *falciparum* malaria [4,5]. Following this, in 2005, the country launched a scaling up of its control and prevention programme with strategies that lead towards malaria elimination in the country [6]. However, since, there is lack of pre-elimination data of malaria parasite population circulating in the country, it was found important to study the genetic diversity, using the two highly polymorphic markers, merozoite surface protein 1 and merozoite surface protein 2.

Merozoite surface protein 1 and 2 of *P. falciparum* are major blood-stage malaria vaccine targets [7] and are also suitable markers for the identification of genetically distinct *P. falciparum* parasite sub-populations. MSP1 is major surface protein of approximately 190-kDa size. It plays a major role in erythrocyte invasion [8] and is a major target of immune responses [9]. MSP1 contains 17 blocks of sequence flanked by conserved regions [10] Block 2, which is the most polymorphic part of MSP1, is grouped into three allelic families namely K1, MDA20, and RO33 type [11]. MSP2 is glycoprotein consisting five blocks where the central block is the most polymorphic [12]. MSP2 alleles are grouped into two allelic families, FC27 and 3D7/IC1.

Based on the literature, there is no published data available on the diversity and multiplicity of infection in uncomplicated falciparum malaria in the country. Such data have importance in documenting the parasite genetic diversity changes due to elimination pressure through the scaled up malaria control intervention. This study was under taken to collect background data on the extent genetic diversity of *P. falciparum* in the pre-elimination period from Kolla-Shele in southwestern Ethiopia.

## Methods

### Study area

**Figure 1:**
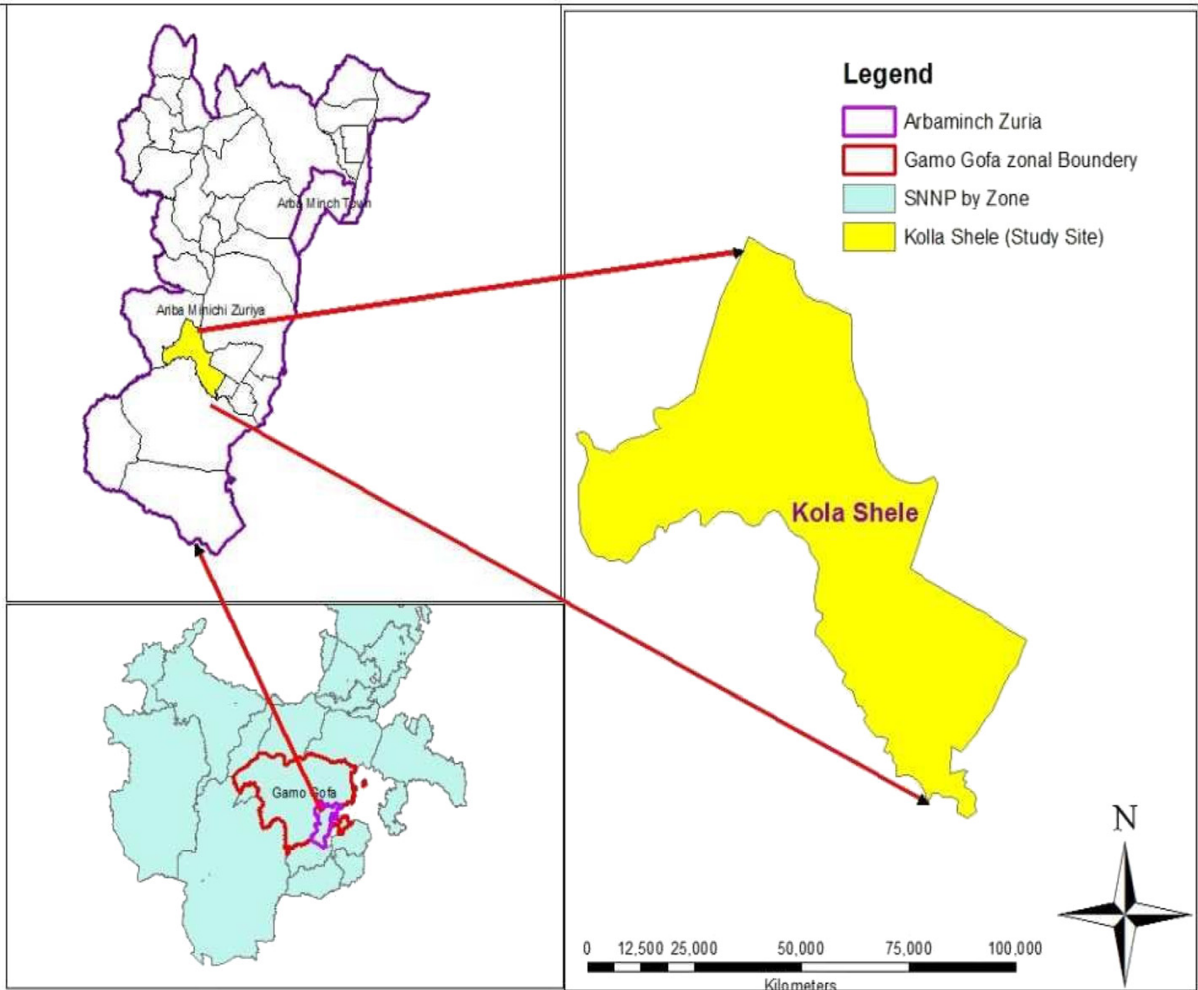
**Map of study area, Kolla-Shele in southern Ethiopia (Source: [** 13 **]).**

The study was conducted in Kolla-Shele area, Arbaminch Zuria District, southern Ethiopia. Where malaria transmission is highly seasonal and markedly unstable. The study area is located about 27 kms from Arbaminch town and 532 kms from Addis Ababa. Kolla Shele is one of the kebeles of Arbaminch Zuria ditrict, with a catchment population of 47,044 inhabitants (Figure 1) [13]. This area lies between 1,250 and 1,400 metres above sea level (asl). The study area has an entomological inoculation rate (EIR) of 17.1 infectious bites per person per year [14]. *Plasmodium falciparum* is the predominant parasite species and *Anopheles arabiensis* is the major vector.

### Study population and blood sample collection

A total of 88 *P. falciparum* blood spotted samples were collected from patients aged six months to twenty years, who visited the health centre during the evaluation of a therapeutic efficacy study, September-December, 2008 [15]. The patients who were febrile, with an axillary temperature ≥37.5°C, were positive for asexual *P. falciparum,* and were residents within the catchment area (i.e. 5–10 km radius of the health centre) were enrolled if they consented, in accordance with the inclusion criteria [16]. Finger prick blood samples were collected on day 0 when patients were enrolled in the study. The blood was spotted onto filter paper (Whatman® 927 mm), air-dried and stored at −20°C, for analysis at the Parasitology Molecular Laboratory of the Ethiopian Public Health Institute (EPHI).

### Extraction of parasite DNA

Genomic DNA was extracted from the stored dried blood spots (collected on Day0) using Chelex-100® (Bio-Rad Laboratories CA) method [17], giving a final volume of 200 μl for each sample. All parasites DNA extracted were stored at -20°C until used for the amplification reaction.

### Allelic typing of Plasmodium falciparum msp1 and msp2 genes

All samples were genotyped for *P. falciparum* using the nested polymerase chain reactions (PCRs) technique [18]. All reactions were carried out in a final volume of 25 μl containing 20 mM dNTP, 10 μM of each primer, and 1 unit /25 ul reaction volume of Taq DNA polymerase (Roche Applied Science, Germany). In the first round reaction, 4 μl of genomic DNA was added as a template. In the nested reaction, 2 μl of the first PCR product was added. Each amplification profile consisted of initial denaturation at 94°C for 3 minutes, followed immediately by 30 cycles at 94°C for 1 min; 50°C for 35 seconds, and 68°C for 2.5 minutes. The final cycle had a prolonged extension at 72°C for 3 minutes. PCR reaction mixtures were incubated in a thermal cycler (MyCycler-BioRad, Hercules, USA). Allelic specific positive control 3D7 and DNA-free negative controls were included in each set of reaction [19]. Gel photographs were re-scored by visual comparison of DNA fragments and for individual samples, alleles were identified according to band size [Additional file 1: Figure S1]. The size of the PCR products was estimated using 100 bp DNA ladder marker (Boehringer Mannheim Marker VI). The size polymorphism in each allelic family was analysed, assuming that one band represented one amplified PCR fragment derived from a single copy of *P. falciparum msp-1* or *msp-2* genes. Alleles in each family were considered the same if fragment sizes were within 20 bp interval [20]. The heterozygosity index (He), was calculated by using the following formula: H_e_ = [n/(n-1)] [(1-Σpi2 )], where n is the number of isolates sampled and pi is the allele frequency at a given locus [21].

### Data analysis

Data were entered and analysed using SPSS version 16 (SPSS Inc., Chicago, IL, USA). The *msp1* and *msp2* allelic frequency was calculated as the proportion of the allele found for the allelic family out of the alleles detected in the isolates. The number of samples with more than one amplified fragment within total samples is defined as frequency of polyclonal infection. The mean multiplicity of infection (MOI) was calculated as a total number of *P. falciparum* genotype detected per total number of positive samples. The Chi-square test was used to compare MOI with the event of a previous malaria attack. Spearman’s rank correlation coefficient was calculated to assess association between MOI and parasite density and age. Statistical significance was defined as *P* value ≤ 0.05.

### Ethical clearance

The study was ethically approved by the Institutional Review Board (IRB) of Department of Medical Laboratory Science Addis Ababa University. In addition, Scientific and Ethical approval was obtained from Scientific and Ethical Review Office (SERO) of the Ethiopian Public Health Institute (EPHI).

## Results

### Demographic and parasitological data

The characteristics of the study populations are detailed in Table 1. The patients ages ranged from six months to 20 years (mean age: 7.5 years). The geometric mean parasite density was 7,702 (95% CI: 5839.9-10156.4) parasites/μl of blood with a range of 1,000 to 100,000 parasites/ μl. The parasite DNA from the 88 *P. falciparum* isolates was analysed for *msp1* and *msp2* genes. The estimated of frequency of *msp-1* and *msp-2* gene amplification reactions with family specific primers was 67% (59/88) and 44% (39/88), respectively.

**Table 1:** **Demographic characteristics of the study subjects** ***at*** **Kolla-Shelle Health Centre, Arbaminch Zuria District, southwest Ethiopia (n = 88)**

**Characteristics of patients**	**Values**
Mean age	7.47 ± 5.3
Age range	6 months to 20 years
Sex ratio ( M/F)	1.1 (45/43)
Geometric mean parasitaemia (parasites/μl)	7,701.5 (5839.9-10156.4).
Parasite density range	1000 to 100,000

### Allelic diversity of *Plasmodium falciparum msp1* and *msp2* genes

Length polymorphism was assessed in 88 *P. falciparum* isolates within the allelic families of *msp1* and *msp2* with a total of 155 distinct fragments detected*.* This allele typing analysis displayed the highly polymorphic nature of *P. falciparum* in Kolla-Shele isolates with respect to *msp1*. In *msp1*, K1, RO33 and MAD20 allele types were identified. Frequencies of different *msp-1* alleles and their combinations and multiplicity of infection are shown in Tables 2 and 3.

**Table 2:** **Allele typing and diversity profiles of** ***Plasmodium falciparum*** **isolates from Southwest Ethiopia based on genetic diversity of msp1 and msp2, Koll-Shele area Southwest Ethiopia, 2014**

**Allelic type**	**n (%)**	**Allelic type**	**n (%)**
MSP-1		MSP-2	
K1	20(33.9)	FC27	4(10.3)
MAD20	5(8.5)	3D7/IC1	8(21.5)
RO33	9(15.2)	FC27 + 3D7/IC1	27(69.2)
RO33 + K1	15(25.4)		
K1 + MAD20	3(5.1)		
RO33 + MAD20	2(3.4)		
Mad20 + K1 + Ro33	5(8.5)		
Total	59		39

**Table 3:** ***Plasmodium falciparum*** **genotypes and base pair ranges observed in** ***msp1*** **and** ***msp2*** **, as well as their respective allelic families, Koll-Shele area, Southwest Ethiopia, 2014**

	***msp-1***			***msp-2***	
	**K1**	**MAD20**	**RO33**	**FC27**	**3D7/IC1**
Base pair range	200-250	100–300	150-225	300-600	200- 500
No. of genotypes	3	3	5	7	5
Total no. of genotypes	11			12	
Overall multiplicity of infection	1.7			1.6	

The proportion of K1, MAD20 and RO33 types were 33.9%, 8.5% and 15.2%, respectively. The remaining nearly 42.5% were the poly-allelic types of *msp1* (K1/MAD20, K1/RO33, MAD20/RO33 and K1/MAD20/RO33). The monoclonal infections were 34 (57.6%). Among Polyclonal infections those that carried two allelic types K1/RO33, K1/MAD20, MAD20/RO33 comprised 25.4%, 5.1% and 3.4%, respectively. Trimorphic infections K1/MAD20/RO33were detected in 8.5% of cases (Table 2)**.**

A total of 23 individual *msp* alleles were identified (11 for *msp1* and 12 for *msp2).* Among *msp1* isolated, three for K1 (200–250 bp), five for RO33 (150–225 bp) and three for MAD20 (100-300 bp) allele families were observed (Table 3). With respect to *msp2*, both FC27and 3D7/IC1 allele types were detected. The frequency of samples having only 3D7/IC1 allelic family (21.5% (8/39)) was higher than the sample with only FC27 allelic type (10.3% (4/39)). Twenty-seven of the isolated (69.2%) carried both *msp2* allelic families (Table 2). On the other hand, among cases that were positive for *msp2* alleles, 31% (12/39) were monoclonal infection while 69.2% (27/39) were polyclonal infections. The length variants of the amplified products were seven for FC27 (300–600 bp) and five for 3D7/IC1 (200–500 bp) (Table 3).

Overall, the mean multiplicity of infection was 1.8 (95% CI: 1.48-2.04). Out of the 88 samples, 52 (59%) harbored more than one parasite genotype. When considering *msp1*and *msp2* genes separately, the overall multiplicity of infection was 1.7 and 1.6, respectively. The heterozygosity index, which represents the probability of being infected by two parasites with different alleles at a given locus, was 0.79 for *msp1* and 0.54 for *msp2* loci.

**Figure 2:**
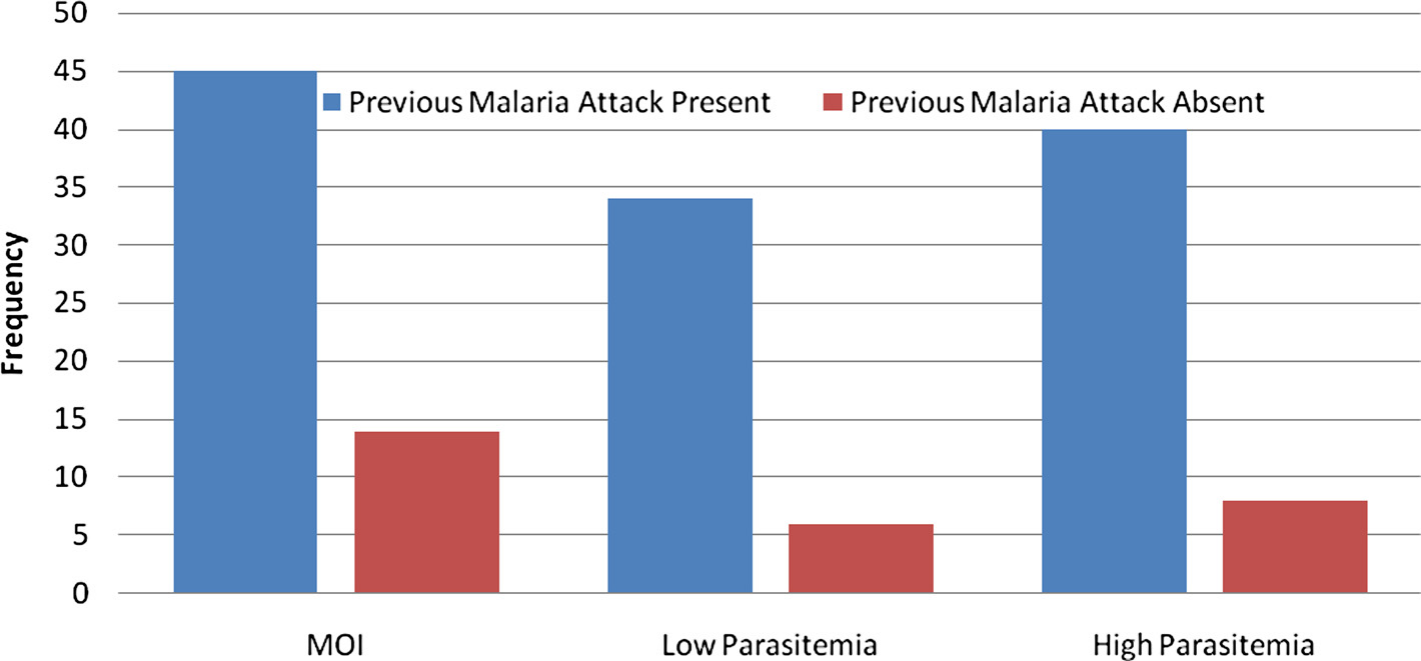
**Comparison of MOI and parasite density against previous malaria attack in Kolla-Shell, Southwest Ethiopia.**

**Figure 3:**
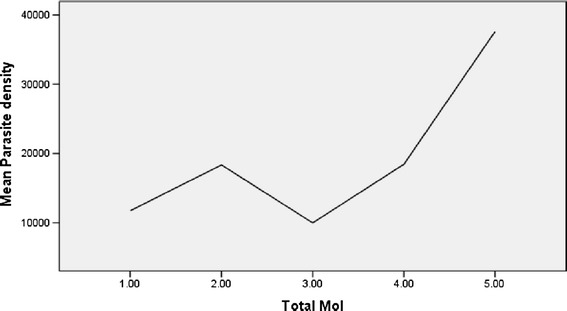
**Relationship between mean parasite density and multiplicity of infection (n = 88).**

A statistically significant correlation between previous malaria attack and MOI was observed (*P* = 0.00), but not non-expose malaria (Figure 2). Neither there was any significant correlation between multiplicity of infection and parasite density of patients (Spearman rank coefficient = 0.03; *P* = 0.8) (Figure 3).

## Discussion

In Ethiopia, less attention has been given to investigating the genetic diversity of *P. falciparum* than other countries. This is the first MSP based study to provide information about the genetic diversity of *P. falciparum* in Ethiopia. These finding may be an important element for implementing malaria control strategies in the country, as elimination may influence genetic diversity and their heterozygosity.

The allele specific *P. falciparum msp1* and *msp2* genotyping has shown that malaria parasite population in Kolla-Shele area is moderate to high allelic diversity. However, the number of alleles may have been underestimated due to the limitation of the technique. Indeed, fragments with the length interval of less than 20 bp could not clearly be distinguished as a separate allele. In their allelic frequency, out of the 59 allelic types detected in *msp1*, the K1 allelic family with 33.9% allelic frequency besides its representation in poly allelic bands, was found predominant This is in line with previous studies in Central Africa, Gabon, Benin, and Ghana [18,22-24], but in contrast to studies from Sudan and Malaysia where RO33 allele was predominant [25,26]. With regard to *msp2*, 39 allelic types were found and the alleles belonging to 3D7/IC1 family were more frequently detected (21.5%). This is similar to data reported from Kenya, Congo Brazzaville and other sub-Saharan-African countries [10,20,27], as well as Peru and Iran [28,29].

The entomologic inoculation rate (EIR) study conducted in the same region showed a value of 17.1 infectious bites per person per year [14], the moderate to high EIR value reported shows the meso-endemicity of malaria transmission which is in agreement with this finding of moderately high genetic polymorphism of 60% and MOI of 1.8. It is reported that the multiplicity of infection in an infected patient may be due to an important entomological inoculation rate as shown in the Senegal where patients are exposed to a large number of infective bites [30].

The Nei unbiased heterozygosity index (H_e_) was high for *msp1* (0.79), indicating a larger genotype diversity within the *msp1* locus than that of *msp2*, with H_e_ 0.54, which is nearly 0.5, i.e. the maximum expected Nei diversity index for allelic locus. This is almost parallel to the Hardy Weinberg’s 0.5 “2pq” equilibrium heterozygosity, in equal frequency P and q alleles. Similarly a moderate heterozygosity level of 0.51 < He 1998-1999-2002 < 0.58 was observed in the population diversity of *P. falciparum* in Djibouti [31]. This, however, indicates only the state of equilibrium heterozygosity at the microsatellite loci, which by 2009 reached a state of total similarity, He = 0, indicating the monoclonality of the Djibouti strain at the studied loci. What will be found in the future with the process of elimination peaking pace in Ethiopia remains to be observed, especially with the highly divergent allelic locus of *msp1*.

The predominant families in the *msp-1* and *msp-2* were K1 and 3D7/IC1, respectively, indicating that both K1 and 3D7/IC1 are good indicators for determination of MOI, at least in Southern Ethiopia. This is because high numbers of bands were encountered with genotypes of these two allelic families. These findings are in agreement with previous study in Republic of Congo [20]. But it is in contrast to studies in Uganda and the Sudan [25,32] where RO33and FC27 allelic families were found predominant.

The FC27 fragment of 400 bp, which was the most prevalent in clinical episodes of malaria among symptomatic malaria children, similar observations were reported from the Republic of Congo and Burkina Faso [33,34]. This may indicate an association between this FC27 allelic type and clinical episode.

This study also showed that the number of parasite genotypes carried by subjects with symptomatic infections was not influenced by age. This is in agreement with previous reports in Republic of Congo and Gabon [20,22]. This may be besides PCR limitations, possibly other factors like comparable level of immune naivety of young and older children in the sample population not to selectively “weed out” certain circulating genotypes.

The MOI values reported in this study was higher than found in countries like Malaysia where the multiplicity of *P. falciparum* infection was 1.37 and 1.20 for *msp1* and *msp2*, respectively [26]. but lower from findings in Côte d’Ivoire, where their MOI was found to be 2.88 *msp2* [35]. This discrepancy may be due to differences in geographical areas and their transmission patterns (differing malaria endemicity level) and also due to differences in sample population determination. Generally, however, the higher the malaria transmission level, the greater becomes the tendency to get a higher MOI and mean number of alleles per locus. Thus in neighbouring Djibouti, *P. falciparum* genetic diversity study showed a decrease of MOI from 1.42 at the peak transmission year in 1999 to 1.12 in 2002 and just 1.0, in 2009, the time the control programme advanced in its pre-elimination phase. With 1.8 MOI, this is nearly two alleles per locus and parallel to this polyclonality, as explained above is observed a moderate (*msp2*) to high (*msp1*) H_e_ values.

The present study found that about two-third of the samples (59%) harboured multiple genotypes; almost similar frequency (62%) to that in the Sudan [25], while 83% of the sample population harboured multiple genotypes**,** in the Republic of Congo [20]. This may go with unstable seasonality of transmission in most malarious areas of Ethiopia, which is more similar with the Sudan than that of West Africa.

The mean MOI of persons with previous exposure to malaria attack is higher compared to persons with absence of previous malaria attack (non-exposed) i.e. high frequency of MOI correlates with high frequency of parasitic density. The finding may indicate that persons with lower parasitic density may have low acquired immunity (higher risk of clinical malaria), that they become symptomatic at a lower parasite threshold, unlike those with previous exposures. This is a tacit indicator that just few previous exposures, can elicit certain level of clinical immunity to make them tolerant to lower parasitaemic threshold up to 10,000Ps/ul blood.

This study represents a first attempt to analyse the molecular characteristic of *P. falciparum* population. However, future study needs to be designed to increase the representative sample sizes in different transmission areas and use more robust techniques, such as microsatellite DNA sequencing, to study in depth the molecular diversity of the *P. falciparum* parasite.

## Conclusion

The results of this study showed genetic diversity and allelic distribution in *msp1* and *msp2* in *P. falciparum* isolates from Kolla-Shelle area. The change in H_E_ and MOI could be potential useful parameters in the evaluation of intervention against malaria, but this needs further studies in several parts of the country, to examine the allelic dominances and the dynamism of spatial/periodic changes in the genetic diversity of *P. falciparum* in Ethiopia.

## Additional file

Additional file 1: Figure S1. Allelic family size polymorphism Using a 50 bp DNA ladder molecular marker (MM) different fragments of base pairs of MSP2 were identified by gel electrophoresis.
