# Supplementary material for: Genetic diversity of Plasmodium falciparum isolates based on MSP-1 and MSP-2 genes from Kolla-Shele area, Arbaminch Zuria District, southwest Ethiopia
Source: Malar J. 2015 Feb 14;14:73. doi: 10.1186/s12936-015-0604-8 (PMC4340489; doi:10.1186/s12936-015-0604-8)
Supplement: Additional file 1: Figure S1. — Allelic family size polymorphism Using a 50 bp DNA ladder molecular marker (MM) different fragments of base pairs of MSP2 were identified by gel electrophoresis. [file 12936_2015_604_MOESM1_ESM.doc]

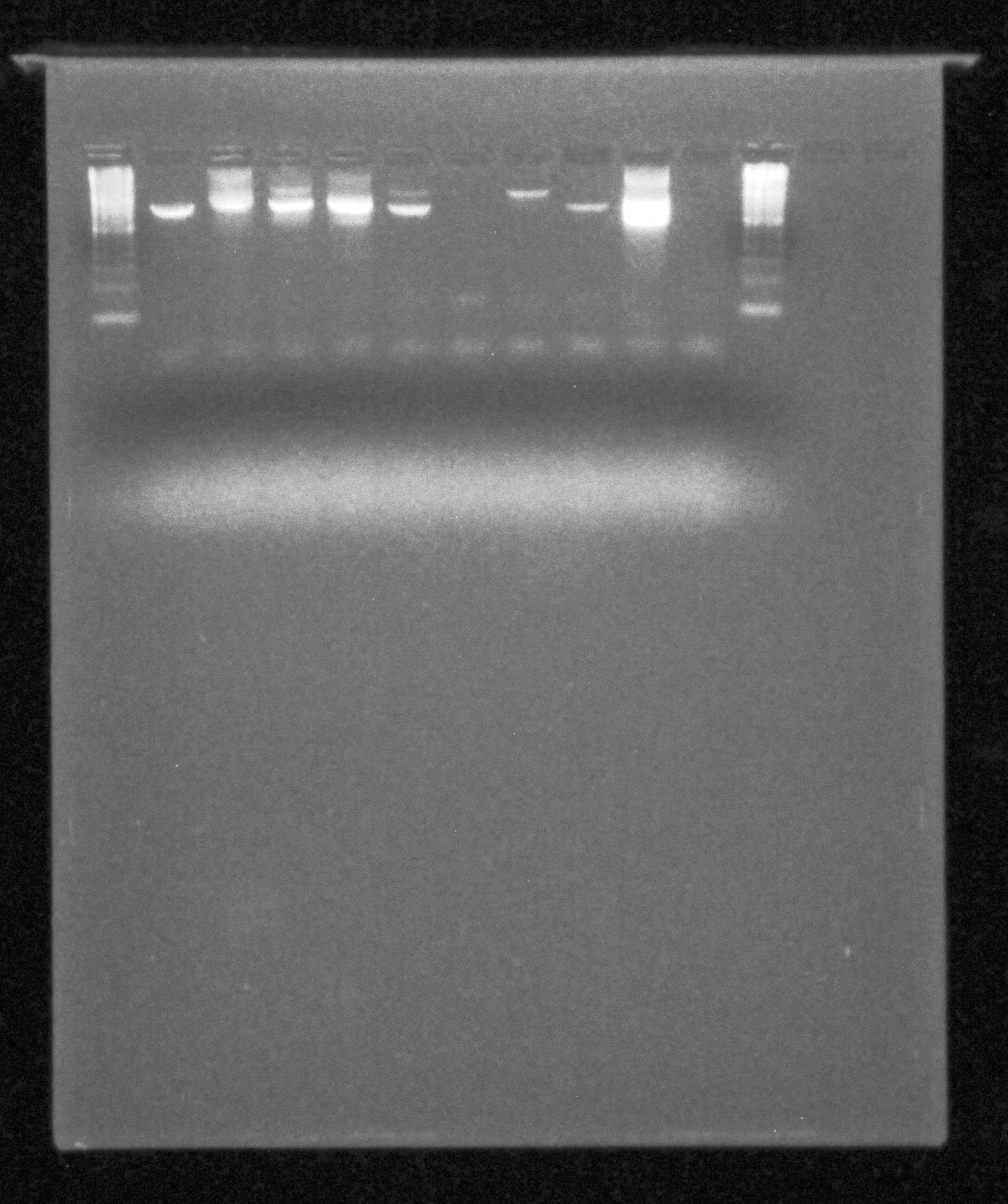


MM 1 2 3 4 5 6 7 8 9 10 MM

**Additional file 1 Figure 1. Allelic family size polymorphism Using a 50 bp DNA ladder molecular marker (MM) different fragments of base pairs of MSP2 were identified by gel electrophoresis.**
